# Rapid Analysis of Visual Receptive Fields by Iterative Tomography

**DOI:** 10.1523/ENEURO.0046-21.2021

**Published:** 2021-12-07

**Authors:** Calvin D. Eiber, Jin Y. Huang, Spencer C. Chen, Natalie Zeater, Alexander N. J. Pietersen, Dario A. Protti, Paul R. Martin

**Affiliations:** 1 Save Sight Institute, The University of Sydney, Sydney, New South Wales 2000, Australia; 2 School of Medical Sciences, The University of Sydney, Sydney, New South Wales 2006 Australia; 3 Australian Research Council Centre of Excellence for Integrative Brain Function, The University of Sydney, Sydney, New South Wales 2000, Australia

**Keywords:** lateral geniculate nucleus, marmoset, receptive field, retina, sensory coding, vision

## Abstract

Many receptive fields in the early visual system show standard (center-surround) structure and can be analyzed using simple drifting patterns and a difference-of-Gaussians (DoG) model, which treats the receptive field as a linear filter of the visual image. But many other receptive fields show nonlinear properties such as selectivity for direction of movement. Such receptive fields are typically studied using discrete stimuli (moving or flashed bars and edges) and are modelled according to the features of the visual image to which they are most sensitive. Here, we harness recent advances in tomographic image analysis to characterize rapidly and simultaneously both the linear and nonlinear components of visual receptive fields. Spiking and intracellular voltage potential responses to briefly flashed bars are analyzed using non-negative matrix factorization (NNMF) and iterative reconstruction tomography (IRT). The method yields high-resolution receptive field maps of individual neurons and neuron ensembles in primate (marmoset, both sexes) lateral geniculate and rodent (mouse, male) retina. We show that the first two IRT components correspond to DoG-equivalent center and surround of standard [magnocellular (M) and parvocellular (P)] receptive fields in primate geniculate. The first two IRT components also reveal the spatiotemporal receptive field structure of nonstandard (on/off-rectifying) receptive fields. In rodent retina we combine NNMF-IRT with patch-clamp recording and dye injection to directly map spatial receptive fields to the underlying anatomy of retinal output neurons. We conclude that NNMF-IRT provides a rapid and flexible framework for study of receptive fields in the early visual system.

## Significance Statement

We present new means to characterize rapidly the linear and nonlinear properties of receptive fields in early stages of visual processing. We analyze light-evoked response properties using new tomographic methods developed for medical imaging. The tomographic method is rapid, can be used to characterize many cells simultaneously, and reveals detailed structure of receptive field organization in monkey and mouse visual system.

## Introduction

Visual signal processing is the single largest activity of the human brain; by some estimates, over half of primate neocortex receives visual signals (Felleman and Van Essen, 1991). But what is the nature and purpose of these visual signals? Since the first descriptions of visual receptive fields in single-neuron recordings from the eye of horseshoe crabs (Hartline, 1938), two main conceptual models for answering this question have been developed. One model (Marr, 1982; Poggio et al., 1985) describes visual receptive fields as linear spatial filters that send undifferentiated messages from the eye to the brain, where they can be refined and analyzed. The alternative model (Lettvin et al., 1959; Barlow, 1972) describes receptive fields as nonlinear feature detectors, which are selectively triggered by relevant features in the visual environment and feed specific pathways for visually-guided behaviors. Visual function depends however on the simultaneous integration of visual signals from many cells, including cell types well-characterized by linear spatial filter models (Kuffler, 1953; Rodieck and Stone, 1965) as well as cells better described by trigger-feature models (Hubel and Wiesel, 1961; Barlow and Levick, 1965; Riesenhuber and Poggio, 2002). This practical (and, arguably, theoretical) inconsistency points to a need for methods which can characterize linear and nonlinear receptive fields within the same framework.

Attempts to unify analyses of linear and nonlinear receptive fields have included methods based on responses to spatiotemporal noise, using the principle of reverse correlation (De Boer and Kuyper, 1968; De Valois et al., 1979; Jones and Palmer, 1987; Brown et al., 2000; Chichilnisky, 2001; Liu et al., 2017). The profound suppressive effects of inhibitory circuits at the first stages of visual processing in the retina however largely restrict such pseudo-random techniques to characterizing the linear kernel of visual responses. Further, there is a need for stimuli and analysis techniques which can robustly activate cell ensembles comprising spatially distributed receptive fields. To accomplish these goals, receptive field mapping using flashing bars and the inverse radon transform has been demonstrated in the retina (Johnston et al., 2014; Stincic et al., 2016) and primary visual cortex (Katona et al., 2012; Nauhaus et al., 2016). Here, we advance these approaches by articulating the radon transform with iterative reconstruction tomography (IRT) algorithms adapted from the field of tomographic image analysis (Andersen and Kak, 1984; Hansen and Jørgensen, 2018). The IRT method reveals detailed features of receptive field organization in the precortical visual system, and allows rapid analysis of linear and nonlinear receptive fields mapped for many cells simultaneously under a single experimental framework.

## Materials and Methods

### Extracellular recordings

Extracellular recordings were made from the lateral geniculate nucleus (LGN) of six adult marmosets (*Callithrix jacchus*, four male, two female) using high-impedance single electrodes and multielectrode arrays (NeuroNexus); 42 isolated single units included 11 parvocellular (P) cells, 11 magnocellular (M) cells, and 20 koniocellular (K) cells. Because K cells comprise heterogenous sub-populations (for review, see Hendry and Reid, 2000; Martin and Solomon, 2019), they were further classified according to their specific response properties. They comprised eight color-coding (blue/yellow-selective) K cells (Szmajda et al., 2006), seven K-on/off cells (Eiber et al., 2018), and five K cells with other response properties. Procedures were approved by the institutional animal care and ethics committee at the Author University. Anesthesia and analgesia were maintained by continuous intravenous delivery of Sufentanil citrate (6–30 μg kg^−1^ h^−1^; Sufenta Forte, Janssen) and inspired 70:30 mix of N_2_O and carbogen. At the conclusion of the experiment, the animal was overdosed with pentobarbitone sodium (80–150 mg kg^−1^, i.v.) and positions of recorded cells were recovered histologically.

### Patch-clamp recordings

*In vitro* whole-cell patch-clamp recordings of retinal ganglion cells (RGCs) were performed in whole-mount retina from dark-adapted young adult male mice (C57Bl/6J, *n* = 15). Surgical procedures in mice were conducted under low-light conditions using infrared or dim red illumination. Animals were anesthetized by isoflurane (Pharmachem), then euthanized by cervical dislocation. Eyes were removed and the retina was dissected in carboxygenated Ames medium (Sigma-Aldrich) and transferred to a recording chamber. During recording, the recording bath was perfused with 36°C carboxygenated Ames medium. Whole-cell patch-clamp recordings of RGCs took place under an Axioskop microscope (Zeiss) using infrared light, with a K^+^-gluconate-based intracellular solution containing Lucifer yellow (0.2%). At the end of each experiment retinae were fixed with 4% paraformaldehyde in 0.1 m phosphate buffer for 30 min then processed using anti-Lucifer yellow antibodies (1:10,000, Invitrogen) to reveal cell morphology. Most of the recorded cells (12/15) were classified as type A (Sun et al., 2002; Bleckert et al., 2014), having large cell bodies with radiating branching dendrites; the remainder were classified as type C6/J-RGC (3/15), having an asymmetric comet-like dendritic field (Kim et al., 2008; Liu and Sanes, 2017). Cell morphology was captured using a Leica SPE-II confocal microscope and images were stitched and processed to align the traced dendritic morphology to the location of the cell during recording using ImageJ, Adobe Photoshop and Illustrator. Dendritic fields were traced in MATLAB using the maximum-intensity projections of the dendritic field images; information regarding the stratification of the RGCs was discarded.

### Visual stimulus

Visual stimuli (flashing bars) were generated using custom visual stimulus software (EXPO, Peter Lennie) at six different orientations and 21 positions per orientation, typically using 1- to 2-s presentations flashed at 5 Hz in the LGN and 1–2 Hz in the retina. For a subset of marmoset LGN recordings, cone-selective stimuli were generated as described previously (Brainard, 1996; Tailby et al., 2008) using the spectral radiance distribution of the monitor phosphors, the spectral sensitivity of marmoset short-wave sensitive (S) and medium/long (ML) wave-sensitive cone photoreceptors, and knowledge of the spectral absorbance of the optic media and macular pigment (Tailby et al., 2008). Three replicates of this stimulus procedure were presented; all stimuli were presented in pseudo-random order. In most cases, there was no obvious difference between receptive field maps computed from a single replicate compared with the analysis of all three replicates. In both mice and marmosets, flashing bar recordings were complemented by more traditional recordings of drifting grating responses to stimuli of varying contrast and spatial frequency, as well as responses to flashed spots of various sizes. For retinal recordings, stimuli were presented at intensity of 0.25 cd/m^2^ on a dark background using a white OLED monitor (SVGA, 800 × 600 pixels, refresh rate: 60 Hz, eMagin Corp, white point CIE [x, y] [0.32, 0.33]). For LGN recordings, stimuli were presented on a gray background (mean luminance 50 cd/m^2^) using a LED monitor (VIEWPixx, Vpixx Technologies, refresh rate 120 Hz).

For cells recorded in the LGN, the contrast response function and temporal responses to flashed spots were used to classify each recorded cell as either P, M, or a subclass of K cell, as described in the results. For the majority of LGN cells (which lack significant S-cone input), we found little difference between receptive fields mapped with achromatic stimuli versus ML-cone-isolating stimuli. Receptive field analyses for these cells are based on achromatic stimuli if available, and ML-cone-isolating stimuli otherwise, as described below. For purposes of statistical comparison, drifting grating responses in a reference population of P cells (*n* = 130) and M cells (*n* = 90) were drawn from a larger database of recordings conducted under similar recording conditions. To control for the influence of eccentricity on the computed receptive field statistics, this dataset was reduced to an eccentricity-matched set of 69 P cells and 69 M cells.

Results were mildly dependent on the choice of bar width and temporal frequency; bar width and temporal frequency were set based on pilot experiments to give the best compromise between response amplitude, data acquisition time (which is generally reduced by broader bars) and spatial resolution (which is improved by narrower bars). For example, we found that mapping RGC receptive fields with broader bars (90 vs 30 μm) and faster stimuli (5 vs 1 Hz) tended to produce larger estimates of receptive field diameter. In a three-way ANOVA controlling for variation between cells (*n* = 74 stimulus sessions), increasing bar size increased estimated diameter (*p* = 0.001) but the effect of temporal frequency was not significant (*p* = 0.072). Where tested, changing spatial and temporal parameters did not change the overall visual appearance of the receptive field map, nor the location of the maximum response, nor the presence of transitions from positive to negative spatial weights. The choice to stimulate using six orientations was made following Johnston et al. (2014), who found that five orientations analyzed through filtered back-projection were sufficient to estimate the center, size, shape, and orientation of the receptive field center, and that additional projections did not increase the amount of spatial information that could be extracted. Under a reanalysis of our data including only three orientations we could identify the receptive field center and basic organization in 28/42 cells (66.67%). When three rather than six bars were used for reconstruction, star-shaped streak artifacts (described further below) were more pronounced and we had difficulty reconstructing non-Gaussian receptive field structures such as annular or displaced binocular receptive fields.

### Response analysis

**Figure 1.**
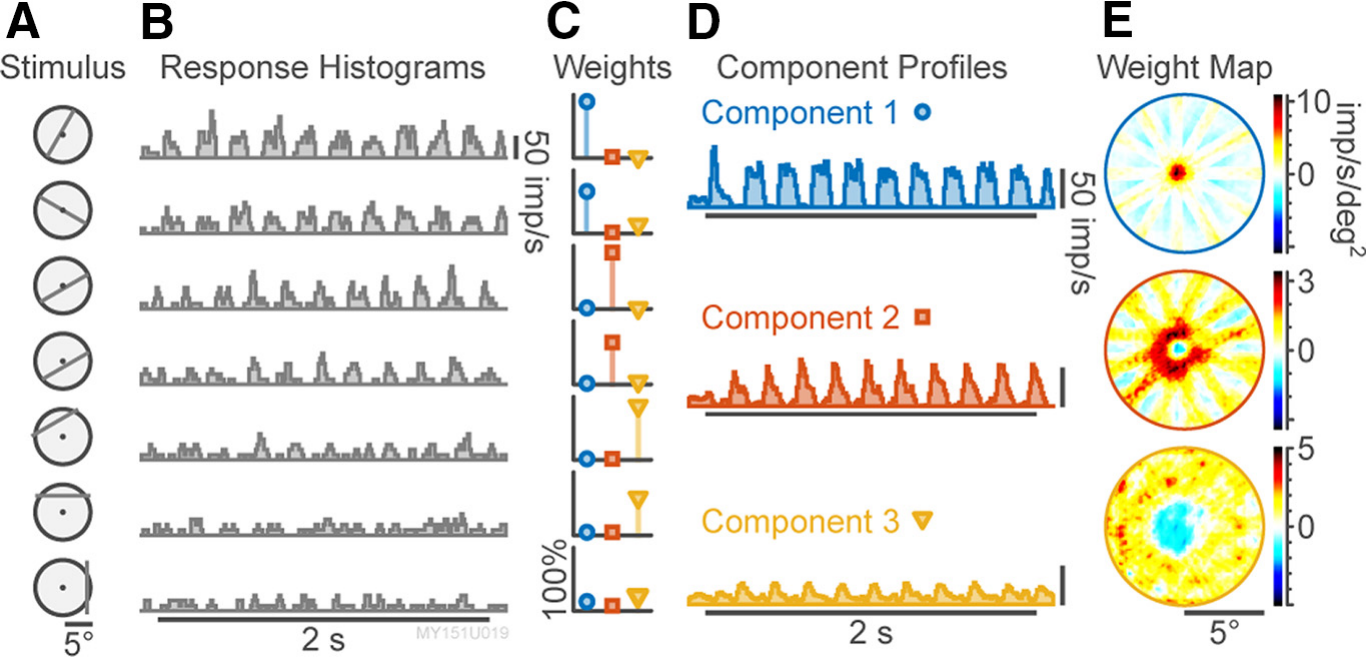
Schematic example of tomographic analysis. ***A***, Example stimulus locations (achromatic flashing bar, 5 Hz) presented to a M-on cell (10.3° eccentricity) in marmoset LGN. ***B***, Peristimulus histograms (PSTHs) to 2 s of stimulation at 5 Hz. Horizontal bar shows stimulus onset and duration. ***C***, Computed NNMF weights for each bar position, normalized to 100% per-weight maximum, corresponding to the PSTHs shown in ***B***. ***D***, Response component profiles from NNMF analysis, corresponding to center and surround mechanisms. In order to emphasize the non-negativity of the NNMF output, prestimulus baselines were not subtracted from the components displayed. ***E***, Receptive field maps of the component weights shown in ***C***. Negative values indicate inhibitory contributions to the spatial summation for that component.

Extracellularly recorded action potentials were discriminated by on-line and off-line principal component analysis (Expo; Blackrock offline spike sorter, Plexon); intracellular recording action potentials were determined using simple threshold-crossing procedure. Spike trains were analyzed offline using MATLAB (R2015a; MathWorks). For each cell, a maximally descriptive feature set of temporal profiles and spatial weights was constructed from the individual trial spike responses using non-negative matrix factorization (NNMF). The NNMF decomposition of the time-varying responses R to stimulus is given by

(1) $$R_{\left[i,t\right]}≅P_{\left[t,k\right]}W_{\left[k,i\right]},$$

where $R_{\left[i,t\right]}$ is a matrix of the individual response peristimulus time histograms (PSTHs), containing the instantaneous spike-rate at time t as driven by the i ^th^ stimulus, which can be represented as the product of a t×k matrix of up to k excitatory temporal profiles $P_{\left[t,k\right]}$ and a k×i matrix of weights $W_{\left[k,i\right]}$ representing the extent to which the response to the i ^th^ stimulus matches profile $P_{1}$ … $P_{k}$. An example of this decomposition is shown in Figure 1*B–D*, which show the individual response PSTHs $R_{\left[i,t\right]}$ (Fig. 1*B*), the computed weights $W_{\left[k,i\right]}$ (Fig. 1*C*), and the temporal profiles $P_{\left[t,k\right]}$ (Fig. 1*D*). The NNMF decomposition of the response follows the same form as a principal component analysis where the primary differences P and W are constrained to be non-negative. Responses to cone-isolating stimuli were analyzed together to generate common temporal profiles but distinct spatial weights for the i^th^ S-cone versus the i^th^ ML-cone stimulus.

Because of the non-negativity constraints on the temporal profiles, the approach outlined above only captures excitatory components of the receptive field structure. In order to recover the inhibitory components of the center and surround, the non-negativity constraint on the temporal component of the wave profiles was relaxed according to

(2) $$R_{\left[i,t\right]}≅P_{\left[t,k\right]}W_{\left[k,i_{s}\right]}=\left(PF^{-1}\right)\left(FW\right),$$

where the rotation matrix F (dimension $k_{max}$) has entries of 1 on the diagonal and nonnegative values elsewhere. Equation 2 allows for negative values in the rotated response components $\left(PF^{-1}\right)$, while preserving the rotated NNMF component weights (FW) as positive values. In order to generate approximately unimodal (spatially compact) spatial weights, response weights were rotated to fit Gaussian profiles as given by

(3) $${\left(\mathrm{FW}\right)}_{k}=g_{k}e^{-{\left(\frac{d_{i}}{4r_{k}}\right)}^{2}}+b_{k}.$$

Where the $k^{th}$ rotated response weight ${\left(\mathrm{FW}\right)}_{k}$ is a scaled Gaussian function of distance from the receptive field center with height $g_{k}$ . The distance from the $i^{th}$ stimulus to the receptive field center is $d_{i}$, the radius of the $n^{th}$ component is $r_{n}$, and the baseline activation of the n^th^ component is $b_{n}$. For a simple two-component center-surround response, this model has 10 free parameters: two for the off-diagonal entries of *F*, two for the receptive field center coordinate, and a radius, amplitude, and baseline level for each component. Optimum values of parameters W and F in Equation 3 were estimated in MATLAB using constrained nonlinear least-squares minimization with the off-diagonal entries of F (Eq. 3) initialized to 0. Equation 3 provides a parametric estimate of the receptive field diameter, which was adjusted to account for the finite bar-width of the flashing-bar stimulus, and can be compared with traditional measurements of receptive field diameter using drifting gratings at different spatial frequencies. Example data and MATLAB scripts implementing the NNMF-IRT analysis and receptive field visualization are available at https://github.sydney.edu.au/ceiber/rapid-RF-analysis.

### Selection of number of NNMF components

In order to determine the number of NNMF components to analyze, temporal profiles and mapped spatial weights were visualized for a range of choices of $k_{max}$. The number of components was increased until either (1) additional components generated temporal profiles which did not change from prestimulus to stimulus on visual inspection, or (2) additional components primarily separated responses to stimuli of different orientations, as opposed to response components with distinct temporal profiles. When responses were over-segmented in this way, the NNMF analysis produced inconsistent sinograms which could not be spatially reconstructed. For consistency, all data were analyzed with $k_{max}\geq 2$.

### Receptive field reconstruction

The inverse radon transform is a general inverse problem, where a solution to the linear system y=A x is sought. The measured data $y={\left[y_{1}...y_{s}\right]}^{T}$ are the average component weights from Equations 1, 2 for each stimulus 1...s,which are given by the matrix product of A, an s×p matrix of the stimulus where each row is the image corresponding to the i^th^ stimulus, and x, a p×1 vector of the point-wise strength of the receptive field at each point in visual space. This problem is ill-posed when the dimension of the receptive field map (number of pixels p) is greater than the number of measurements s. This problem can be solved with the simultaneous algebraic reconstruction technique (SART; Andersen and Kak, 1984; Hansen and Jørgensen, 2018), using the normalized cumulative periodogram stopping rule (Hansen et al., 2006; Rust and O’Leary, 2008). For comparison, filtered back-projections were computed for the receptive field component weights using a first-order low-pass Butterworth filter with a normalized corner frequency of 0.8 cycles following reference (Johnston et al., 2014). The SART algorithm is one of a family of fast simultaneous iterative reconstruction algorithms; comparison showed negligible difference in reconstruction performance between SART and other similar algorithms (Hansen and Jørgensen, 2018; data not shown). For chromatic stimuli, receptive fields were computed from the spatial weights of the S-cone-isolating and ML-cone-isolating stimuli independently.

### Experimental design and statistics

Receptive field parameters estimated from the NNMF-IRT Gaussian fits given by Equation 3 were compared with estimates based on responses to drifting gratings of varying spatial frequency. The standard difference-of-Gaussians (DoG) model (Rodieck and Stone, 1965; Enroth-Cugell and Robson, 1966) was used to analyze the first harmonic (f1) response of the drifting grating response:

(4) $$K=\pi k_{c}r_{c}^{2}e^{-{\left(\pi r_{c}\omega \right)}^{2}}-\pi k_{s}r_{s}^{2}e^{-{\left(\pi r_{s}\omega \right)}^{2}},$$

where the response spike rate K is a function of the strength of the center and surround (given by $k_{c}$ and $k_{s}$) and the radius of the center and surround (given by $r_{c}$ and $r_{s}$), for an input stimulus ω (in cycles/degree). The ratio of surround to center gain is $k_{s}/k_{c}$. For purposes of comparison, first harmonic (f1) responses were estimated from the NNMF component profiles in LGN cells; a comparison of the fitted parameters for drifting grating and flashing bar stimuli for P and M cells is shown in the results, Figure 5. All comparison statistics are based on two-sided Wilcoxon rank-sum tests for independent samples (unless otherwise stated) and are corrected for multiple comparisons using the Holm–Bonferroni method.

## Results

### Matrix factorization of flashing bar responses

Responses were collected *in vivo* from extracellular recordings of single units in the LGN of marmosets (*C. jacchus*, *n* = 6 animals); 42 isolated single units included 11 P cells, 11 M cells, and 20 K cells. Responses were also collected *in vitro* in patch-clamp recordings from whole-mount retina of dark-adapted mice (*n* = 15 cells in 15 animals). The visual stimuli were stationary flashing (square-wave modulated) bars, presented at 5 Hz in the LGN and 1–2 Hz in the retina, for 1–2 s per stimulus. The stimulus set comprised bars at 6 different orientations and 21 positions per orientation. Using three replicates of this set of stimuli presented in pseudo-random order, complete receptive field maps could be collected for single-cell or array recordings in under 5 min per replicate. Figure 1*B* shows sample responses to three replicates of achromatic flashing bar stimuli for a typical LGN M-on cell.

PSTHs (Fig. 1*B*) were constructed for each replicated trial. NNMF was applied to decompose the spike-rate into temporal profiles (Fig. 1*D*) and corresponding spatial weights (Fig. 1*C*). These spatial weights were mapped to form a receptive field image using IRT (Fig. 1*E*). Previous work which used white-noise stimuli and a similar reconstruction approach required 60- to 180-min recording time (Liu et al., 2017). In common with principal component analysis (Schwartz et al., 2006), NNMF yields a low-dimensional representation of the response as a sum of k independent components (see Materials and Methods, Selection of number of NNMF components). Unlike principal component analysis, the non-negativity constraints mean that NNMF yields a sparse representation of independent response elements (Ding et al., 2005), and is mildly tolerant of nonstationarities in the recorded data. We limited analyses to decompositions of two or three components, as demonstrated in Figure 1*C–E*. Structure could sometimes be observed in mapped spatial weights corresponding to additional components, but these were not systemically investigated further. In common with previous approaches (Johnston et al., 2014) receptive field maps for the first two NNMF components generated using IRT show weak “streak” artefacts; the origin and impact of these artefacts is discussed further below.

The M-on cell receptive field center mechanism dominates the first NNMF component, appearing as a phase-locked response in the component PSTH (Fig. 1*D*, upper) and as a small roughly circular region on the IRT weights map (Fig. 1*E*, upper). The second component PSTH comes in opposite phase to the first component (Fig. 1*D*, center) and appears as an annular region in the IRT weight map (Fig. 1*E*, center). The second NNMF component thus shows the excitatory contribution of the surround to spiking responses. The third NNMF component comes in phase with the surround (Fig. 1*D*, lower) within a spatially broad region (Fig. 1*E*, lower) which likely corresponds to the extra-classical suppressive field (Solomon et al., 2002). These data show that the NNMF-IRT analysis can cleanly separate well-characterized components of concentric antagonistic receptive fields in marmoset LGN.

### Simultaneous receptive field mapping

**Figure 2.**
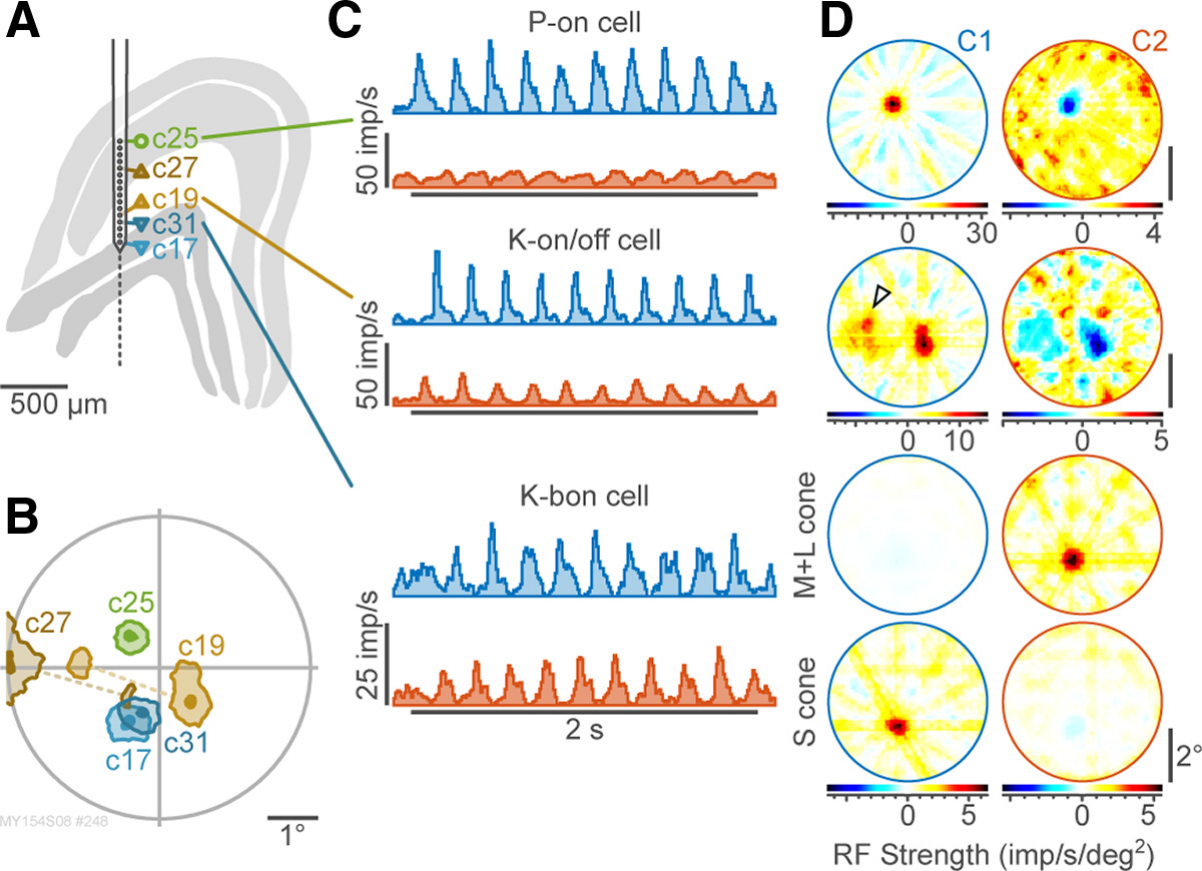
Geniculate array recording. ***A***, Reconstructed LGN and electrode track, showing the location of 16 channels relative to the layers of the LGN. Two K blue-on cells, two K-on/off cells, and one P-on cell were recorded from this site. ***B***, Contour plot of simultaneously recorded RFs for these cells to cone-isolating stimuli, showing outlines at 90% and 50% of the peak response amplitude (filled/shaded areas). ***C***, NNMF component profiles for three example cells; prestimulus baselines were not subtracted. Horizontal bar: stimulus onset and duration. ***D***, Receptive field maps corresponding to the components shown in ***C***. For the K-on/off cell a displaced hot-spot (attributable to weak excitatory input from the nondominant eye) is indicated with an open arrowhead. For the K blue-on cell, spatially coextensive inputs are evident for S-cone-isolating and ML-cone-isolating stimuli.

We next show that the NNMF-IRT procedure can be used to map receptive fields from cells recorded simultaneously through semiconductor array electrodes. Figure 2*A* shows the reconstructed positions of five LGN cells (one P-on cell, two K-on/off cells, and two K blue-on cells) in response to cone-isolating stimuli. Half-maximal and 90% sensitivity contours for first NNMF components of these cells (Fig. 2*B*) show expected visuotopic progression and receptive field dimensions (White et al., 1998). Responses of the P-on cell (Fig. 2*C*,*D*, upper) are dominated by the excitatory contribution of the receptive field center. The K-on/off cell (Fig. 2*C*,*D*, center) received weak excitatory binocular input from the nondominant eye, resulting in a displaced hot-spot in the receptive field map (Fig. 2*D*, arrowhead). This observation is consistent with our previous report of binocular inputs to K cells (Zeater et al., 2015). The K blue-on cells (Fig. 2*C*,*D*, lower) showed opposite-sign responses to S-cone isolating and ML-cone isolating stimuli. These response patterns are consistent with responses to drifting, cone-isolating gratings in single-cell recordings (Eiber et al., 2018). The results show the NNMF-IRT analysis can map the spatiotemporal and chromatic inputs to simultaneously recorded linear and nonlinear receptive fields, with data acquisition time a fraction that required for traditional grating-based analyses. Importantly, responses to individual stimuli were not strongly suppressed by nonspecific activation of suppressive surround mechanisms, which is a chief limitation of approaches where much of the visual field is activated simultaneously (as in, for example, full-field stimulation or pseudorandom checkerboard stimulation).

### Quantitative analysis of spatiotemporal receptive fields

**Figure 3.**
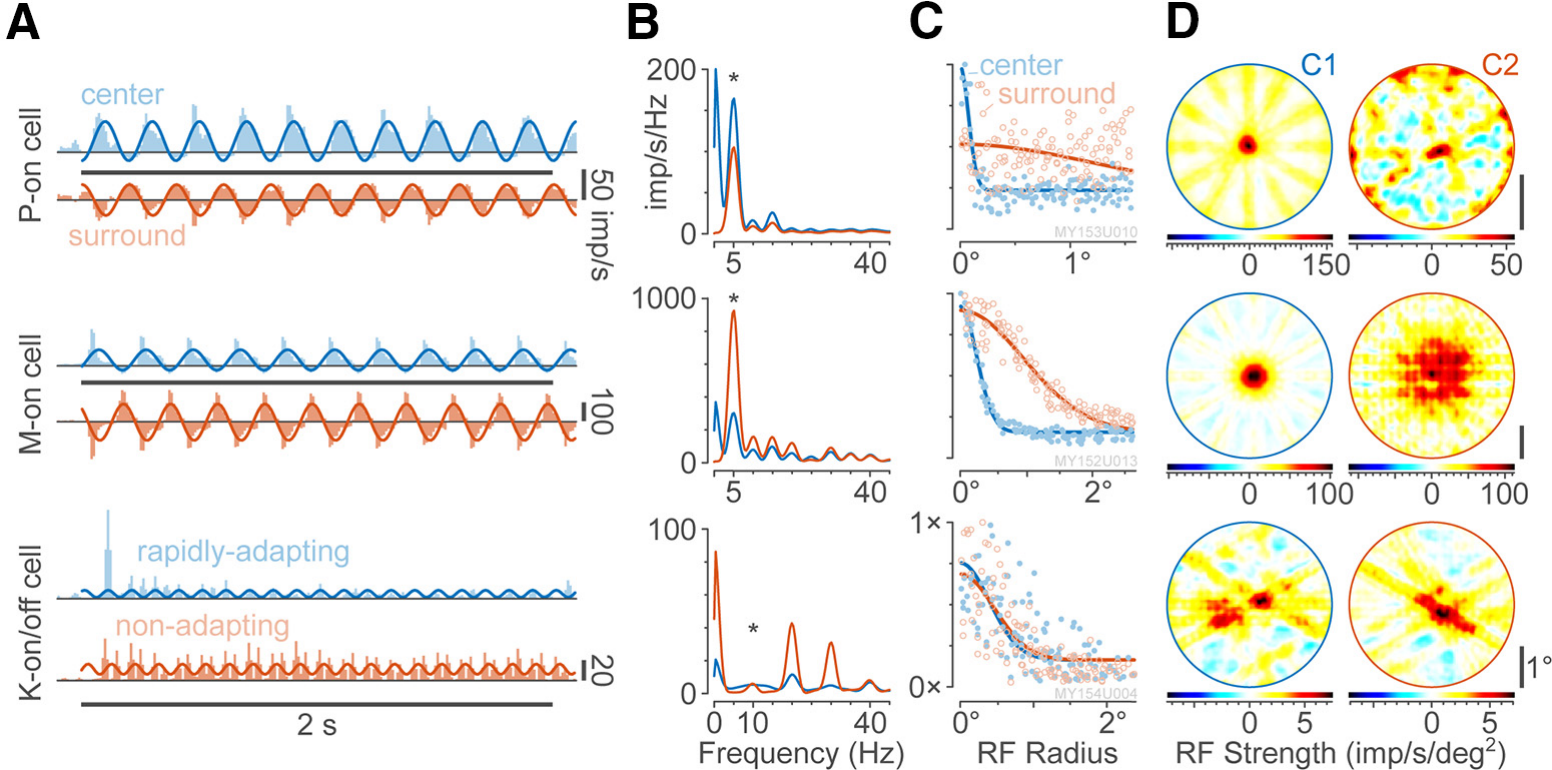
Analysis of geniculate receptive field structure. ***A***, NNMF component profiles for three example cells recorded in marmoset LGN in response to achromatic flashed bars. Upper–lower, P-on cell, M-on cell, K-on/off cell (respective eccentricity 1.6°, 14.6°, and 29.2°). Each row shows one example cell. Sine waves show the response at the stimulus frequency (5 Hz for the P and M cell; 10 Hz for the K-on/off cell). For the P and M cell, the two components capture the responses of the center and the surround; for the K-on/off cell, the two components capture a rapidly-adapting and a nonadapting components of the response. ***B***, Response spectra for the computed NNMF components (red and blue). Asterisk shows stimulation frequency. ***C***, Scatterplot of (normalized) component weights versus distance from the receptive field center. Gaussian fits are shown as thick lines. Each point represents one stimulus (bar) presentation. ***D***, Receptive field maps for the two components. A surround component can be localized for the M cell but not the P cell, and the spatial map for the K-on/off cell demonstrates spatially co-extensive on+off input.

The NNMF approach can be extended to visualize inhibitory receptive field components, as opposed to sums of purely excitatory components. When we transformed the generated spatial weights to best approximate unimodal Gaussian curves (see Materials and Methods; Eq. 2), the corresponding *temporal* response profiles had negative values, representing inhibitory inputs to the receptive field (Fig. 3*A*; the reader should note that this example cell is not the same cell as shown in Fig. 2*C*). In this way, the NNMF approach can be used to bridge between different perspectives regarding receptive field organization. Receptive field radii were computed from the resulting (Gaussian) rotated weights (Fig. 3*C*), and these weights can be mapped using IRT to show the resulting receptive field structure (Fig. 3*D*). For linear LGN cells, this recombination yields the expected center and surround components (example P cell, Fig. *3*, upper; example M cell, Fig. 3, center). The surround profile is substantially more prominent for M cells than for P cells, likely in consequence of the characteristic high contrast gain of M cells (Derrington and Lennie, 1984; Kaplan and Shapley, 1986). For M cells (but not P cells), we also observed bar positions that gave frequency-doubled responses (data not shown) similar to those evoked by flickering counter-phase gratings (Crook et al., 2008).

In addition to characterizing linear receptive fields, the NNMF-IRT analysis can probe receptive fields of highly nonlinear cells. For example, on applying NNMF-IRT to responses of a K-on/off cell, the first NNMF component captures a rapidly adapting component which responded preferentially to the first bar presentation (Fig. 3*B*, lower). The second component captured a nonadapting component which responded equally well across all stimulus presentations in the 2 s period. Both components show evidence of frequency-doubling. For this cell, the two components map as overlapping excitatory input fields (Fig. 3*C*,*D*, lower). As expected (Eiber et al., 2018), the overall extent of the receptive field for this K-on/off cell is broader than that of either the P or M cell receptive field center.

**Figure 4.**
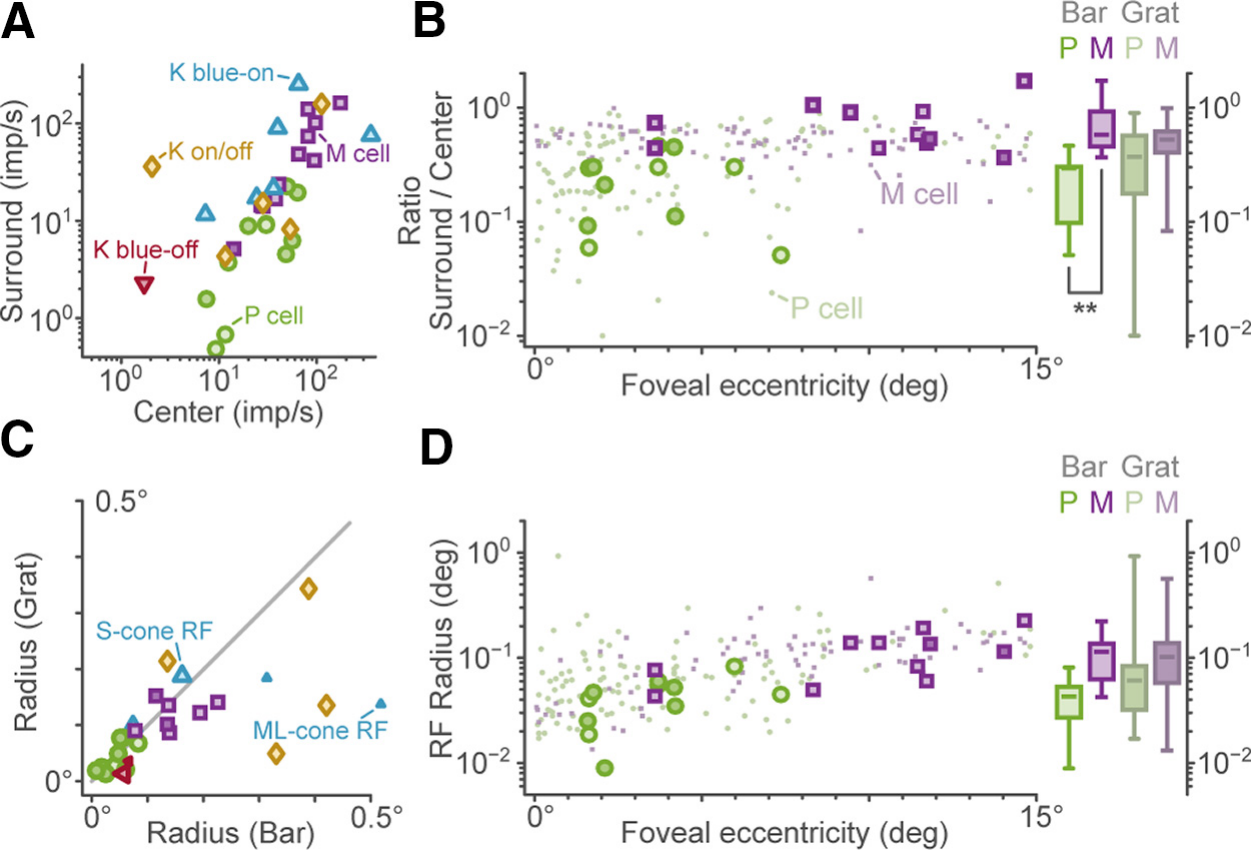
Summary statistics for visual cells in the LGN. ***A***, Observed receptive field center and surround strength. ***B***, Ratio of surround gain to center gain versus eccentricity, measured with bars (large markers) and drifting grating stimuli (small markers). Box charts (right) show the range, median, and intraquartile range of gain ratio for the flashing-bar and drifting-grating stimuli, **p < 0.001. ***C***, Correlation between receptive field center radii (Eq. 3) measured with drifting grating stimuli or flashing bars. ***D***, Receptive field center and surround radius versus eccentricity, as measured using flashing bars and drifting gratings.

Observed differences between P cells and M cells are supported by population statistics (Fig. 4). Measured using flashing bars, P cells had a mean receptive field center radius of 0.04 ± 0.02° (*N* = 11) and our population of M cells had a larger mean receptive field center radius of 0.12 ± 0.06° (*N* = 11, *p* < 0.001). Recorded P cells have a significantly lower ratio of surround to center gain, compared with M cells (*p* < 0.001; population mean ± SD for P cells 0.24 ± 0.15 imp s^−1^ vs 0.75 ± 0.40 for M cells; Fig. 4*A*). These data are quantitatively comparable to data obtained using drifting gratings in a large eccentricity-matched sample of P and M cells (Fig. 4*B*). For K blue-on and K blue-off cells, the relative gain of S-cone to ML-cone inputs was tightly correlated (*r* = 0.94, *p* = 0.002, *n* = 7), with a population mean ratio of 1.17 ± 0.61. Estimated NNMF-IRT spatial properties were likewise correlated with counterpart parameters measured in the same cells using drifting gratings (Fig. 4*C*); for example, center radii derived from NNMF-IRT and gratings were closely correlated, *r* = 0.84 (*p* < 0.001). Receptive field radii could also be calculated using NNMF-IRF for a small number of highly nonlinear K-on/off cells (*n* = 3; Fig. 4*A*,*C*). In sum, these data show that NNMF-IRT yields estimates of spatial receptive field properties with accuracy at least as high as measured using traditional grating stimuli. Further, data acquisition time under the NNMF-IRT method (under 5 min per replicate, see Materials and Methods) is much more rapid than under traditional grating stimuli. For example, the spatial and orientation tuning measurements summarized in Figure 4 required acquisition times >40 min per cell for drifting grating stimuli. This time benefit is further increased by the capacity of NNMF-IRT to characterize simultaneously cells with spatially separated receptive fields (as shown by the example recording in Fig. 2).

### Comparison to underlying anatomy

**Figure 5.**
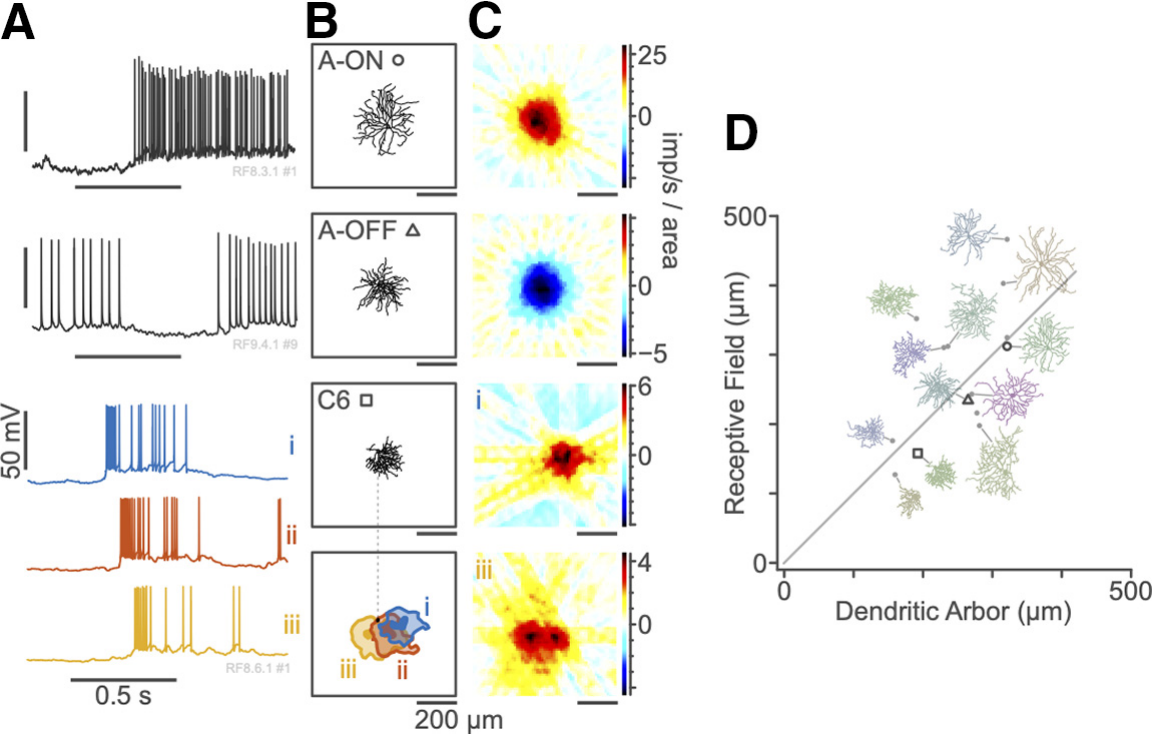
Example mouse RGC responses. ***A***, Representative traces for stimuli intersecting the receptive field center. From top to bottom, an A2 ON cell, an A2 OFF cell, and a C6 cell. Horizontal bar shows stimulus onset and duration. Time courses of the first three NNMF components (***i***, ***ii***, ***iii***) are shown separately for the C6 cell. ***B***, Dendritic morphology of these cells. Bottom panel shows spatial profiles for components (***i***, ***ii***, ***iii***) of the C6 cell. ***C***, Receptive field maps for these cells. Receptive field strength is given in imp/s per 100 × 100 μm^2^; 1 pixel = 12 μm^2^. Lower two panels show maps for components ***i***, ***iii*** of the C6 cell. ***D***, Relationship between dendritic field diameter and measured receptive field diameter for our sample of mouse RGCs. Morphology of traced RGCs shown at 1:10 scale.

Directly correlating neural structure to neural function is important for improved understanding the origins of visual receptive fields. We therefore compared receptive fields mapped with NNMF-IRT to the underlying anatomy of RGCs in mouse retina, as previously demonstrated by Brown et al. (2000) using spatiotemporal white noise. Example intracellular recordings of flash bar responses for three RGCs (Fig. 5*A*) are shown together with reconstructed dendritic morphologies (Fig. 5*B*), shown at the same scale as the spatial weight map of the first NNMF-IRT component (Fig. 5*C*). In common with the linear P and M cells, we recorded in marmoset LGN (Figs. 1-3), the first NNMF-IRF component captures the linear center mechanism of the A-type receptive fields (Fig. 5, upper rows). In contrast, the class C6/J-RGC cell response (Fig. 5*A*, lower) shows substantial nonlinearity. Here, the first three NNMF components capture excitatory responses with distinct latency differences. The weight maps of the first three components are spatially offset, in the same progression as the latency offsets (Fig. 5*B*, lower). This example is consistent with the known selectivity of C6/J-RGC cells for downward retinal image slip (Kim et al., 2008; Liu and Sanes, 2017), but a more extensive study of direction selectivity is beyond the scope of the present study. Across recorded cells, the anatomically measured dendritic field correlated closely with the diameter of the physiological receptive field extracted from the first NNMF-IRT component (*r* = 0.621, *p* = 0.018; Fig. 5*D*).

**Figure 6.**
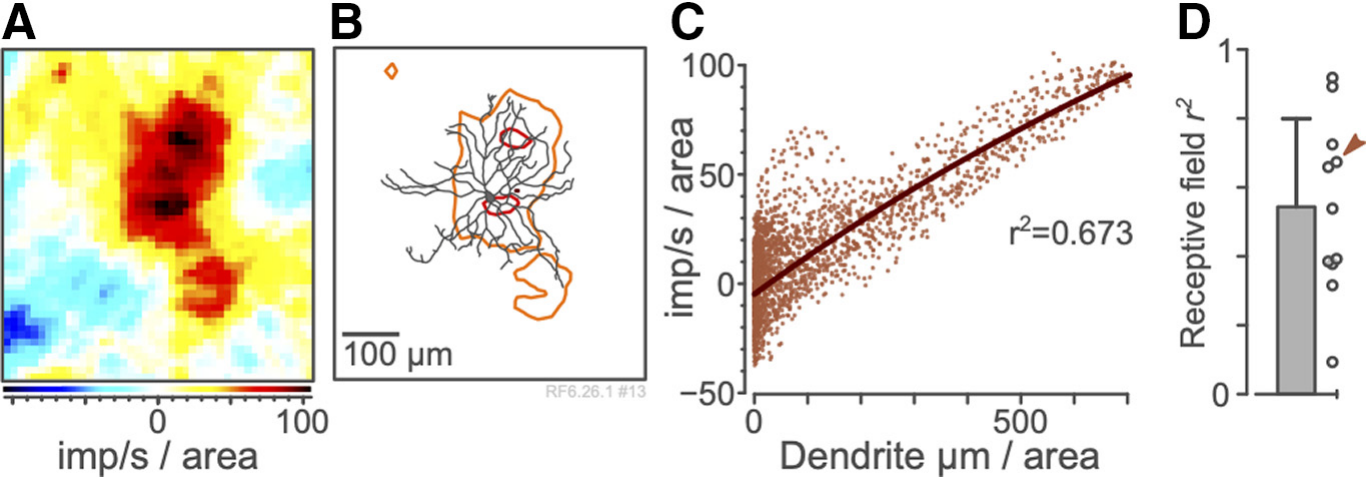
Example and summary statistics for correlation between physiology and anatomy. ***A***, Receptive field map for an A-on RGC, in imp/s per 100 × 100 μm. ***B***, Contour plot of receptive field showing outlines of 90% (red outline) and 50% (orange outline) of the peak response relative to the cell dendritic morphology. ***C***, Correlation between receptive field strength and dendrite density, at 1 point per 12 μm^2^, showing fitted relationship between physiological and anatomic data. ***D***, Summary *r*^2^ (coefficient of determination) values across the population of RGCs. Arrowhead indicates cell shown in ***A–C***. Error bar shows standard deviation.

The local structure of the NNMF-IRF spatial weight map (Fig. 6*A*) showed mild to strong correlation with the local dendritic field density of recorded RGCs (Fig. 6*B*,*C*), after accounting for the lateral spread of signal (estimated half-height radius 54.7 μm) induced by the presynaptic bipolar and amacrine cell circuitry. Across recorded cells the mean *r*^2^ correlation of anatomic to physiological measures was 0.543 ± 0.256 (Fig. 6*D*). In sum, these results show the potential of NNMF-IRT analysis for fine-grained analysis of structure-function relationships.

## Discussion

Here, we build on receptive field mapping techniques using flashing bar stimuli (Johnston et al., 2014) by combining NNMF with IRT. This NNMF-IRT combination allows simultaneous evaluation of linear and nonlinear receptive fields, such as those of direction-selective C6/J-RGCs (Fig. 5) and K-on/off LGN cells (Fig. 3), and can be applied to characterize many receptive fields in parallel. Because each location in the stimulus field is probed independently by a high-contrast bar, the spatial and temporal contributions of weak presynaptic inputs to receptive field can be measured (Fig. 6). The IRT method (Andersen and Kak, 1984) preserves the spatial structure of such weak inputs while reducing the influence of measurement noise, and permits incorporation of prior knowledge into the receptive field reconstruction process (as shown in Fig. 3).

The fine structure of RGC receptive fields is driven by branching patterns of RGC dendrites in the inner plexiform layer of the retina (Brown et al., 2000), with branch density manifest as subunits in the computed receptive field (cf. Brown et al., 2000; Eiber et al., 2018; Turner et al., 2018). The NNMF-IRT method offers a way forward for mapping receptive field subunits, because the spatial resolution of the flashing bar maps can be increased independently of bar contrast. The correspondence of receptive fields mapped using the NNMF-IRT method with receptive fields mapped using complementary techniques such as reverse correlation of spatiotemporal noise (De Boer and Kuyper, 1968; De Valois et al., 1979; Jones and Palmer, 1987; Brown et al., 2000; Chichilnisky, 2001; Liu et al., 2017) will be important to substantiate or refute the utility of the NNMF-IRT method.

A well-established advantage of NNMF for analyzing non-negative inputs such as spike rates is the ease with which the resulting components can be interpreted. The non-negativity constraint acts to bring correlated responses together by increasing the sparsity (fraction of zero or near-zero weights) of the receptive field representation. Subunit-based analyses (Liu et al., 2017; Turner et al., 2018) share this advantage, and both approaches stand in contrast to a strict orthogonal basis vector representation, as would be generated by singular value decomposition (SVD). The effect on the NNMF-derived receptive field maps of response correlations arising from activation of multiple subunits is however not yet known, and requires more research.

We found that NNMF usually led to two or three (very rarely, four) components which could be interpreted in terms of both the temporal response profile (showing a distinct response to stimulation) and the spatial response map (showing a clear center or center-surround structure). We also tested SVD as a tool for response decomposition but found that SVD rarely led to more than a single interpretable response component (data not shown). When higher-order SVD components had a coherent spatial structure, they had an unintelligible temporal structure, and vice-versa. We predict that additional NNMF components will be useful for analyzing receptive field subunit structure at spatial resolution greater than that presented here (Turner et al., 2018).

One limitation that IRT shares with the filtered back projection method is the presence of star-shaped streak artefacts in the receptive field map (Johnston et al., 2014). Our pilot reconstructions of synthetic receptive fields (data not shown) indicate that streak artifacts can be reduced by measuring at more orientations, albeit at the cost of increased acquisition time. Minimizing such receptive field artifacts is particularly important for characterizing nonlinear receptive fields such as those in smooth monostratified RGCs in primates (Rhoades et al., 2019) and loom-detecting RGCs in mouse retina (Katz et al., 2016). Another limitation of our present NNMF-IRT analysis approach was that we were unable to disentangle the excitatory and inhibitory/suppressive components of the receptive field surround in a model-agnostic manner; future work will concentrate on finding better ways to separate these physiologically distinct mechanisms.

We conclude that the NNMF-IRT combination offers a flexible method to isolate receptive field inputs for both linear and nonlinear visually responding cells. As with the filtered back-projection method, the mapping procedure is very rapid – accurate receptive field maps can be constructed from as little as 5 min of data acquisition. This efficiency offers the possibility to measure receptive fields before and after pharmacological manipulations, and to explore contributions of local synaptic processing to receptive field properties in vision. The combination of NNMF and IRT provides a new tool for studying retinogeniculate projections and synaptic signal transformations underlying visual receptive field organization. More broadly, the combination of NNMF and IRT offers an avenue to unify linear (systems-theory) and nonlinear (feature-detector) descriptions of cells in the early visual system.

## Synthesis

Reviewing Editor: Siegrid Löwel, University of Goettingen Decisions are customarily a result of the Reviewing Editor and the peer reviewers coming together and discussing their recommendations until a consensus is reached. When revisions are invited, a fact-based synthesis statement explaining their decision and outlining what is needed to prepare a revision will be listed below. The following reviewer(s) agreed to reveal their identity: Katrin Franke. Note: If this manuscript was transferred from JNeurosci and a decision was made to accept the manuscript without peer review, a brief statement to this effect will instead be what is listed below.

The authors present a significant methodological advance to reconstruct spatial receptive fields of neurons in the pre-cortical visual system. Although no novel scientific insights are presented, the work described will be of interest to the visual neuroscience community. The authors suggest that the NNMF/IRT approach can be used to reliably map visual RFs with relatively little recording time - representing an important improvement compared to many state-of-the-art techniques. A second important advantage is that, by combining with non-negative matrix factorization, non-linear components of the receptive field, such as the silent surround, can also be assessed. The authors validate the method convincingly with electrophysiological recordings from the retina of mice and lateral geniculate nucleus of primates.

The manuscript is prepared to a very high standard. It is written concisely and, for the most part, clearly.

Nevertheless, the authors should address/clarify the following concerns/issues:

1. “What does one gain when using this method compared to others?” The authors should address this question more directly throughout the paper. The study nicely shows that the approach can be used to map RFs of visual neurons, identifying cellular properties that have recently been described using other methods. One big advantage we see is that this works with relatively little recording time which is critical for a number of experimental paradigms. If the authors agree, they should highlight this point more explicitly in the Introduction and the Abstract. In addition, they should provide more information throughout the Results section when comparing their approach to others (e.g. Fig. 4) - how much longer were recording times for traditional moving grating stimuli compared to NNMF/IRT?

2. In addition, the strong focus in the Introduction and Abstract on the fact that their approach is model-agnostic and works for both simple, center-surround and more complex RFs is not very convincing, as many other models/approaches are suitable to recover both simple and more complex RFs. Therefore, we suggest to highlight the point above and down-tune the model-agnostic characteristic of their approach.

3. Many visual neuroscientists study motion and/or orientation and direction selectivity. It would be important to discuss whether this approach is also suitable for studying these properties of visual neurons.

4. The authors mention several times throughout the paper, that the NNMF/IRT approach can be used to map RFs of neuron ensembles and analyze many cells simultaneously (Significance Statement line 30, should be “analyze” instead of “record”?). It is not clear to us why this is highlighted - as far as we understand, the analysis is performed on single cell basis. For example, line 264 states that it can be used to map receptive fields from simultaneously recorded cells - but why should this not be possible? Or do the authors mean that population recordings are often more noisy, so corresponding RFs are more difficult to map? Please clarify.

5. It is unclear when the authors used cone isolating stimuli and how this data was analyzed. Was this presented only during marmoset recordings? And were RFs in response to S vs L/M cone isolating stimuli mapped separately or together? We suggest to clarify in the Results section and also add more details to the Methods.

6. The distinction between excitatory and inhibitory/suppressive surround is unclear. For example, in Fig. 1D both excitatory and inhibitory surround fire in phase and out of phase with the center, but one is excitatory and one suppressive? Does suppressive mean relative to baseline activity? We suggest the authors add a clarification in the Results section and the Methods. In the following, the authors should always say excitatory or suppressive surround when talking about surround to be clearer.

7. Results lines 278 and following: It is unclear what was done here. As far as we understand, their approach maps RFs without any assumptions about their linearity and shape. Is it correct that here, the authors reconstruct the RFs given the NNMF components assuming Gaussian center-surround RFs and compare the result to the agnostic method introduced before? We think it would help to be more explicit here.

8. The motivation for comparing the mapped RFs with the cells’ morphologies is not clear. We suggest the authors clarify in the respective Results section. Also, what do the results tell us about the approach compared to other methods? In the Discussion, the authors conclude that their approach allows to map weak inputs and refer to Fig. 5 - how do the results from Figure 5 show this? We would also then mention in the Results section of the linked Figures.

9. Finally, please clarify whether code and data will be made available upon publication. As the authors present a new approach, this would help potential users a lot!

Minor points:

1. Introduction line 44: We would suggest to phrase more conservatively.

2. Introduction lines 50-52: Remove brackets.

3. Introduction line 64: I suggest to add 1-2 sentences about topographic image analysis.

4. Methods line 114: Remove “.” and add space.

5. Methods line 117: What kind of monitor/projector was used for visual stimulation of mouse experiments?

6. Methods lines 185-190: Unspecific; we suggest to add what criterions were used specifically for 1) and 2). For example, what does “the subdivided components approximately sum to the original component” mean? Also, please refer to Methods in lines 246-251 of the Results section.

7. Results lines 230-232: The authors should explain what 5 Hz bar presentation for 1-2 seconds means - as far as we understand, several bars were then presented at the same time, with varying onset/offset times.

8. Results lines 269-271: Has this feature been described for this cell type previously? If so, please cite here.

9. Results lines 292-300: The conclusion is not obvious from the figure. The second component of the cell looks more transient to us, as it has two peaks where the first component has one. Please clarify.

10. Results lines 317 and following: Fig. 5 labels are wrong.

## Author Response

COMMENT: 1. “What does one gain when using this method compared to others?” The authors should address this question more directly throughout the paper. The study nicely shows that the approach can be used to map RFs of visual neurons, identifying cellular properties that have recently been described using other methods. One big advantage we see is that this works with relatively little recording time which is critical for a number of experimental paradigms. If the authors agree, they should highlight this point more explicitly in the Introduction and the Abstract. In addition, they should provide more information throughout the Results section when comparing their approach to others (e.g. Fig. 4) - how much longer were recording times for traditional moving grating stimuli compared to NNMF/IRT?

RESPONSE: We agree with the reviewer’s point, and have correspondingly changed the title to “Rapid analysis of visual receptive fields by iterative tomography”. We have revised the introduction text (P3, P4) as follows, and have also revised relevant parts of the results (P16):

(P3,4) “Attempts to unify analyses of linear and non-linear receptive fields have included methods based on responses to spatiotemporal noise, using the principle of reverse correlation (De Boer and Kuyper, 1968; De Valois et al., 1979; Jones and Palmer, 1987; Brown et al., 2000; Chichilnisky, 2001; Liu et al., 2017). The profound suppressive effects of inhibitory circuits at the first stages of visual processing in the retina however largely restrict such pseudo-random techniques to characterizing the linear kernel of visual responses. Further, there is a need for stimuli and analysis techniques which can robustly activate cell ensembles comprising spatially distributed receptive fields. To accomplish these goals, receptive field mapping using flashing bars and the inverse radon transform has been demonstrated in the retina (Johnston et al., 2014; Stincic et al., 2016) and primary visual cortex (Katona et al., 2012; Nauhaus et al., 2016). This family of receptive field mapping techniques falls into the category of image tomography, that is, the mathematical derivation of images from lower-dimensional sections or sinograms. Here we advance tomographic receptive field mapping by articulating the radon transform with iterative reconstruction tomography (IRT) algorithms adapted from the field of tomographic image analysis (Andersen and Kak, 1984; Hansen and Jørgensen, 2018). The IRT method reveals detailed features of receptive field organization in the pre-cortical visual system, and allows rapid analysis of linear and non-linear receptive fields mapped for many cells simultaneously under a single experimental framework.”

(P16) “Further, data aquisition time under the NNMF-IRT method (under five minutes per replicate, see methods) is much more rapid than under traditional grating stimuli. For example, the spatial- and orientation tuning measurements summarized in Figure 4 required acquisition times greater than 40 minutes per cell for drifting grating stimuli. This time benefit is further increased by the capacity of NNMF-IRT to characterize simultaneously cells with spatially separated receptive fields (as shown by the example recording in Fig. 2).”

COMMENT: 2. In addition, the strong focus in the Introduction and Abstract on the fact that their approach is model-agnostic and works for both simple, center-surround and more complex RFs is not very convincing, as many other models/approaches are suitable to recover both simple and more complex RFs. Therefore, we suggest to highlight the point above and down-tune the model-agnostic characteristic of their approach.

RESPONSE: We have done our best to address this request, as explained in response #1 above.

COMMENT: 3. Many visual neuroscientists study motion and/or orientation and direction selectivity. It would be important to discuss whether this approach is also suitable for studying these properties of visual neurons.

RESPONSE: We added the following sentence to the discussion (P17):

"This NNMF-IRT combination allows simultaneous evaluation of linear and non-linear receptive fields, such as those of direction-selective C6/J-RGCs and koniocellular on/off LGN cells, and can be applied to characterize many receptive fields in parallel.”

COMMENT: 4. The authors mention several times throughout the paper, that the NNMF/IRT approach can be used to map RFs of neuron ensembles and analyze many cells simultaneously (Significance Statement line 30, should be “analyze” instead of “record”?). It is not clear to us why this is highlighted - as far as we understand, the analysis is performed on single cell basis. For example, line 264 states that it can be used to map receptive fields from simultaneously recorded cells - but why should this not be possible? Or do the authors mean that population recordings are often more noisy, so corresponding RFs are more difficult to map? Please clarify.

RESPONSE: In addition to the clarifications to the introduction in response to points 1-2 above, we have added the following sentence to p14:

"Importantly, responses to individual stimuli were not strongly suppressed by non-specific activation of suppressive surround mechanisms, which is a chief limitation of approaches where much of the visual field is activated simultaneously (as in, for example, full-field stimulation or pseudorandom checkerboard stimulation).”

COMMENT: 5. It is unclear when the authors used cone isolating stimuli and how this data was analyzed. Was this presented only during marmoset recordings? And were RFs in response to S vs L/M cone isolating stimuli mapped separately or together? We suggest to clarify in the Results section and also add more details to the Methods.

RESPONSE: In the relevant sections of the methods, we have made the following clarifications:

Section ’Visual stimulus’ (P6) : “For a subset of marmoset LGN recordings, cone-selective stimuli were generated as described previously...” and “For the majority of LGN cells which lacked significant S-cone input, we found little difference between receptive fields mapped with achromatic stimuli vs ML-cone-isolating stimuli; unless otherwise stated, receptive field analyses are based on achromatic stimuli if available, and ML-cone-isolating stimuli otherwise, as described below.”

Section ’Response Analysis’ (P8): “Responses to cone-isolating stimuli were analyzed together to generate common temporal profiles, but distinct spatial weights for the ith S-cone vs the ith S ML-cone stimulus.”

Section ’Receptive field reconstruction’ (P11): “For chromatic stimuli, receptive fields were computed from the spatial weights of the S-cone- and ML-cone-isolating stimuli independently.”

We have clarified in the figure legends for figures 1-3 that figures 1 and 3 present responses to achromatic stimuli, while figure 2 presents responses to cone-isolating stimuli, with corresponding changes to the results.

COMMENT: 6. The distinction between excitatory and inhibitory/suppressive surround is unclear. For example, in Fig. 1D both excitatory and inhibitory surround fire in phase and out of phase with the center, but one is excitatory and one suppressive? Does suppressive mean relative to baseline activity? We suggest the authors add a clarification in the Results section and the Methods. In the following, the authors should always say excitatory or suppressive surround when talking about surround to be clearer.

RESPONSE: We have added the following text to the methods (P9) and discussion (P19):

(P9) “Due to the non-negativity constraints on the temporal profiles, the approach outlined above only captures the excitatory components of the receptive field structure.”

(P19) “Another limitation of our present NNMF-IRT analysis approach was that we were unable to disentangle the excitatory and inhibitory/suppressive components of the receptive field surround in a model-agnostic manner; future work will concentrate on finding better ways of separating these physiologically distinct mechanisms.”

COMMENT: 7. Results lines 278 and following: It is unclear what was done here. As far as we understand, their approach maps RFs without any assumptions about their linearity and shape. Is it correct that here, the authors reconstruct the RFs given the NNMF components assuming Gaussian center-surround RFs and compare the result to the agnostic method introduced before? We think it would help to be more explicit here.

RESPONSE: We have revised this section and tied it to the above point regarding excitatory and inhibitory surround contributions. The revised text (P14) reads:

“The NNMF approach can be extended to visualize inhibitory receptive field components, as opposed to sums of purely excitatory components. When we transformed the generated spatial weights to best approximate unimodal Gaussian curves (see Methods, eq. 2), the corresponding temporal response profiles had negative values, represent inhibitory inputs to the receptive field (Fig. 3A). In this way, the NNMF approach can be used to bridge between different perspectives regarding receptive field organization.”

COMMENT: 8. The motivation for comparing the mapped RFs with the cells’ morphologies is not clear. We suggest the authors clarify in the respective Results section. Also, what do the results tell us about the approach compared to other methods? In the Discussion, the authors conclude that their approach allows to map weak inputs and refer to Fig. 5 - how do the results from Figure 5 show this? We would also then mention in the Results section of the linked Figures.

RESPONSE: We have clarified the motivation for the comparison to the underlying anatomy in the results section as follows (P16):

"Directly correlating neural structure to neural function is important for improved understanding the origins of visual receptive fields. We therefore compared receptive fields mapped with NNMF-IRT to the underlying anatomy of RGCs in mouse retina, as was done by Brown et al. (2000) using spatiotemporal white noise.” and “... the mean r² correlation of anatomical to physiological measures was 0.543 {plus minus} 0.256 (Fig. 6D). These results are comparable to structure-function relations found by other researchers (Brown et al., 2000).”

COMMENT: 9. Finally, please clarify whether code and data will be made available upon publication. As the authors present a new approach, this would help potential users a lot!

RESPONSE:In section “Response Analysis", we have clarified that “Example data and MATLAB scripts implementing the NNMF-IRT analysis and receptive field visualization are available at https://github.sydney.edu.au/ceiber/rapid-RF-analysis .”

Minor points:

COMMENT: 1. Introduction line 44: We would suggest to phrase more conservatively.

RESPONSE: This section of the introduction has been re-written, see response to major point 1.

COMMENT: 2. Introduction lines 50-52: Remove brackets.

RESPONSE: This section of the introduction has been re-written, see response to major point 1.

COMMENT: 3. Introduction line 64: I suggest to add 1-2 sentences about topographic image analysis.

RESPONSE: We have added a sentence about tomographic image analysis:

”...and primary visual cortex (Katona et al., 2012; Nauhaus et al., 2016). This family of receptive field mapping techniques falls into the category of image tomography, the mathematical derivation of images from lower-dimensional sections. Here we advance tomographic receptive field mapping by articulating the radon transform with iterative reconstruction tomography...”

COMMENT: 4. Methods line 114: Remove “.” and add space.

RESPONSE:We have moved the misplaced reference. The revised text reads

“... the sensitivity distribution of marmoset cone photoreceptors, and knowledge of the spectral absorbance of the optic media and macular pigment (Tailby et al., 2008). Three replicates of this stimulus procedure were presented; all stimuli were presented in pseudo-random order. In most cases, there was no obvious difference between receptive field maps computed from a single replicate compared to the analysis of all three replicates.”

COMMENT: 5. Methods line 117: What kind of monitor/projector was used for visual stimulation of mouse experiments?

RESPONSE: We added the following text to the methods (P6):

"For retinal recordings, stimuli were presented at intensity of 0.25 cd/m2 on a dark background using a white OLED monitor (SVGA, 800 x 600 pixels, refresh rate: 60 Hz, eMagin Corp, white point CIE [x, y] [0.32, 0.33]).”

COMMENT: 6. Methods lines 185-190: Unspecific; we suggest to add what criterions were used specifically for 1) and 2). For example, what does “the subdivided components approximately sum to the original component” mean? Also, please refer to Methods in lines 246-251 of the Results section.

RESPONSE: We have added the desired reference to the methods and clarified as follows (P10):

"The number of components was increased until either 1) additional components generated temporal profiles which did not change from pre-stimulus to stimulus on visual inspection, or 2) additional components primarily separated responses to stimuli of different orientations, as opposed to response components with distinct temporal profiles. When responses were over-segmented in this way, the NNMF analysis produced inconsistent sinograms which could not be spatially reconstructed. For consistency, all data was analyzed with k_max{greater than or equal to}2.”

COMMENT: 7. Results lines 230-232: The authors should explain what 5 Hz bar presentation for 1-2 seconds means - as far as we understand, several bars were then presented at the same time, with varying onset/offset times.

RESPONSE: We have clarified this text as follows (P12) :

"The visual stimuli were stationary flashing (square-wave modulated) bars, presented at 5 Hz in the LGN and 1-2 Hz in the retina, for 1-2 s per stimulus. The stimulus set comprised bars at 6 different orientations and 21 positions per orientation. Using three replicates of this set of stimuli presented in pseudo-random order, complete receptive field maps could be collected for single-cell or array recordings in under 5 minutes per replicate.”

COMMENT: 8. Results lines 269-271: Has this feature been described for this cell type previously? If so, please cite here.

RESPONSE: We have added a citation to Zeater et al 2015 who described binocular inputs to K cells including K-on/off cells. The revised sentence reads (P14)

“The K-on/off cell (Fig. 2C,D center) received weak excitatory binocular input from the non-dominant eye, resulting in a displaced hot-spot in the receptive field map (arrowhead, Fig. 2D). This observation is consistent with our previous reports of binocular inputs to K cells (Zeater et al., 2015).”

COMMENT: 9. Results lines 292-300: The conclusion is not obvious from the figure. The second component of the cell looks more transient to us, as it has two peaks where the first component has one. Please clarify.

RESPONSE: We have clarified the description of this observation as follows:

"the first NNMF component captures a rapidly adapting component which responded preferentially to the first bar presentation (Fig. 3B lower). The second component captured a non-adapting component which responded equally well across all stimulus presentations in the 2 s period. Both components show evidence of frequency-doubling”. [We have updated the labels of figure 3 to match this new language.]

COMMENT: 10. Results lines 317 and following: Fig. 5 labels are wrong.

RESPONSE: We have corrected figure 5 to bring the labels into alignment with the text description and legend.
